# Multiple Stressors in the Environment: The Effects of Exposure to an Antidepressant (Venlafaxine) and Increased Temperature on Zebrafish Metabolism

**DOI:** 10.3389/fphys.2019.01431

**Published:** 2019-11-19

**Authors:** Hossein Mehdi, Leslie M. Bragg, Mark R. Servos, Paul M. Craig

**Affiliations:** Department of Biology, University of Waterloo, Waterloo, ON, Canada

**Keywords:** venlafaxine, temperature, metabolism, zebrafish, wastewater, multiple stressors

## Abstract

Aquatic organisms are continuously exposed to multiple environmental stressors working cumulatively to alter ecosystems. Wastewater-dominated environments are often riddled by a myriad of stressors, such as chemical and thermal stressors. The objective of this study was to examine the effects of an environmentally relevant concentration of a commonly prescribed antidepressant, venlafaxine (VFX) [1.0 μg/L], in addition to a 5°C increase in water temperature on zebrafish metabolism. Fish were chronically exposed (21 days) to one of four conditions: (i) 0 μg/L VFX at 27°C; (ii) 1.0 μg/L VFX at 27°C; (iii) 0 μg/L VFX at 32°C; (iv) 1.0 μg/L VFX at 32°C. Following exposure, whole-body metabolism was assessed by routine metabolic rate (RMR) measurements, whereas tissue-specific metabolism was assessed by measuring the activities of major metabolic enzymes in addition to glucose levels in muscle. RMR was significantly higher in the multi-stressed group relative to *Control*. The combination of both stressors resulted in elevated pyruvate kinase activity and glucose levels, while lipid metabolism was depressed, as measured by 3-hydroxyacyl CoA dehydrogenase activity. Citrate synthase activity increased with the onset of temperature, but only in the group treatment without VFX. Catalase activity was also elevated with the onset of the temperature stressor, however, that was not the case for the multi-stressed group, potentially indicating a deleterious effect of VFX on the anti-oxidant defense mechanism. The results of this study highlight the importance of multiple-stressor research, as it able to further bridge the gap between field and laboratory studies, as well as have the potential of yielding surprising results that may have not been predicted using a conventional single-stressor approach.

## Introduction

Pharmaceuticals and personal care products (PPCPs) are frequently introduced and detected in aquatic environments (Daughton and Ternes, 1999; Metcalfe et al., 2010; Arlos et al., 2014, 2015). PPCPs enter aquatic environments from a variety of sources, such as treated municipal and hospital wastewater, as well as agricultural runoff (Daughton and Ternes, 1999; Bottoni et al., 2010). The concentrations of most pharmaceuticals in the surface water of aquatic environments are generally in the ng/L to low μg/L range (Fent et al., 2006; Kümmerer, 2010). However, despite the relatively low concentrations of these chemicals, their impact on aquatic organisms can be significant and has become a source of growing concern. This partly stems from the biochemical nature of these chemicals, as they have the ability to alter the physiological and behavioral responses of exposed aquatic organisms due to the highly conserved drug targets and physiological pathways across vertebrates (Arnold et al., 2014; Brown et al., 2014; Margiotta-Casaluci et al., 2014; McCallum et al., 2017; Mehdi et al., 2018; Saaristo et al., 2018). Another concern of these chemicals that is relatively unexplored is their frequent presence with other stressors, especially in environments impacted by wastewater inputs. Wastewater treatment plant (WWTP) effluent-dominated environments typically suffer high nutrient loads, decreased dissolved oxygen levels, increased water temperature, as well the presence of PPCPs and other chemicals of concern (Chambers et al., 1997; Daughton and Ternes, 1999; Environment Canada, 2001; Kinouchi et al., 2007; Odjadjare and Okoh, 2010; McCallum et al., 2019).

Venlafaxine (VFX), a selective serotonin-norepinephrine reuptake inhibitor (SNRI), is a heavily prescribed and readily detectable antidepressant found in many Canadian waterways that receive WWTP effluents (Metcalfe et al., 2010; Arlos et al., 2014). VFX and its active metabolite, O-desmethyl venlafaxine (O-VFX) are mainly introduced into WWTP effluents via human excretion; approximately 5% of the average human daily dose is excreted in urine as the unchanged parent form and 29% in the active metabolite form (Metcalfe et al., 2010). VFX and O-VFX are often detected at higher concentrations than any other antidepressant drug and its active metabolite in WWTP effluents and effluent-receiving environments (Schultz and Furlong, 2008; Metcalfe et al., 2010). In discharged WWTP effluent, VFX and O-VFX have been detected at concentrations ranging from 808 to 2,050 ng/L and 1,637 to 1,927 ng/L, respectively (Metcalfe et al., 2010; Arlos et al., 2015). Whereas in the surface water of effluent-receiving environments in the Grand River watershed (Southern Ontario, Canada), VFX and O-VFX have been detected at concentrations ranging from 61 to 901 ng/L and 167 to 1,472 ng/L, respectively (Metcalfe et al., 2010). Despite these relatively high concentrations, very little is known about the impacts of this drug on the metabolic responses of aquatic organisms, especially in combination with other stressors that may be present in WWTP effluent-dominated environments.

Temperature, known as the “ecological master factor” is one of the most important determinants of life-cycle events in ectotherms, because of its influence on metabolism, energy production and expenditure, development, survival, and growth (Harig and Fausch, 2002; Lee, 2003; Farrell et al., 2008; Schultz and Bertrand, 2011). Various studies in the past have examined the effects of temperature on the metabolic and energetic responses in fish, however, the effects of such an ecologically relevant stressor have frequently been ignored in the field of ecotoxicology, especially in studies examining the effects of PPCPs on aquatic organisms. This is somewhat surprising, especially since the toxicity of various contaminants has been demonstrated to be enhanced by increasing temperatures, as reviewed in Noyes et al. (2009). Studies investigating the interactive effects of temperature and contaminants are critical, especially since many contaminants of emerging concern (CEC) in treated wastewater effluent are often found in the presence of thermally polluted environments produced via WWTPs. Effluent discharged from WWTPs can be a source of thermal pollution in effluent-receiving environments (Environment Canada, 2001), increasing the temperature by as much as 5–9°C (personal observations downstream of Woodward Avenue WWTP, Hamilton, ON, Canada, 2018). Previous studies have demonstrated links between the effects of temperature on the toxicity of chemicals using a variety of fish species and chemicals (Nussey et al., 1996). Zebrafish (***Danio rerio***) exposed to cadmium at temperatures ranging from 12 to 34°C demonstrated increasing cadmium-tissue accumulation and toxicity, measured by mortality, with increasing temperature (Vergauwen et al., 2013). Similar trends have been observed in ***Penaeus semisulcatus*** exposed to ammonia at different temperatures (Kir et al., 2004), as well as ***Prochilodus scrofa*** exposed to copper at different temperatures (Carvalho and Fernandes, 2006). While there are a number of studies examining the effects of temperature on the toxicity of contaminants, to our knowledge, this phenomenon is largely unexplored in studies examining the effects PPCPs on aquatic organisms. This is especially important as temperature may regulate the uptake of PPCPs by aquatic organisms, thereby, increasing their toxicity.

In our study, we were interested in how increased water temperature and VXF exposure interact and alter fish metabolic responses specifically. Metabolic physiology is an important indicator and contributor to fitness as it is linked to various levels of biological organization. Previous studies have demonstrated that exposure to WWTP effluent poses additional metabolic costs demonstrated by elevation in oxygen consumption rates (Du et al., 2018, 2019; Mehdi et al., 2018). VFX, at environmentally relevant concentrations, has also been shown to potentially act as a metabolic disruptor in fish when exposed to secondary stressors (Best et al., 2014). Added metabolic costs associated with contaminant exposure can result in energy-allocation trade-offs, thereby, potentially affecting fundamental basal processes such as growth, reproduction, and behavior (Scott and Sloman, 2004).

This study aimed to investigate the effects of an environmentally relevant concentration of VFX [1.0 μg/L] in combination with a 5°C increase in water temperature on the metabolic responses of zebrafish. We examined the combined and individual effects of the two stressors on whole-body metabolism by measuring routine metabolic rate (RMR). We also assessed tissue metabolic capacity by measuring muscle glucose and activities of enzymes involved in key metabolic pathways including glycolysis [pyruvate kinase (PK) and lactate dehydrogenase (LDH)], β-oxidation of lipids [3-hydroxyacyl CoA dehydrogenase (HOAD)], aerobic capacity [citrate synthase (CS) and cytochrome c oxidase (COX)], and antioxidant defense capacity [catalase (CAT)].

## Materials and Methods

Adult, mixed sex zebrafish were acquired from a fish wholesale facility (AQUAlity Tropical Fish Wholesale Inc., Mississauga, ON, Canada) and maintained in acrylic tanks (density of <5 fish/L) in a recirculating Habitats^®^ Z-Hab System (Pentair Aquatic Eco-Systems Inc., Apopka, FL, United States). Water supplying the system underwent reverse osmosis, deionization, aeration, biological and chemical filtration, and UV sterilization. Water in the system was maintained at 27°C, pH of 7.5, and conductivity of 670 μS/cm. Fish were kept under a 12h:12h light-dark cycle and fed twice daily. Food consisted of a mixture of ground commercial fish food (TetraMin Tropical Flakes, Blacksburg, VA, United States) and live brine shrimp. This feeding schedule was maintained until start of the exposure experiment. Zebrafish were chosen as an ideal laboratory candidate to assess both the impact of VFX and temperature, with minimal disruption from other stress factors, as they are acclimated to aquarium housing. It should be noted, however, that the responses of lab-reared model organisms may differ from endemic wild organisms. It should also be noted that the responses demonstrated in this study using the tropical/subtropical zebrafish may differ from responses exhibited by temperate species, as fish adapted to different climates may exhibit different responses to temperature. Therefore, proper cross-species comparisons must be considered to solidify our findings. All experimental protocols followed the guidelines of the Canadian Council on Animal Care and were approved by the animal care committee at the University of Waterloo (AUPP #15-03).

### Exposure Design

Adult, male zebrafish were exposed to environmentally relevant concentrations of VFX [1.0 μg/L] with or without an additional thermal stress over a period of 21 days. This exposure period was chosen because it has been demonstrated that steady-state is reached within 14–28 days in fish following a temperature change (Sidell et al., 1973; Hazel and Carpenter, 1985). Twenty fish were placed in 12-L aquaria, with three tank replicates per treatment. Each tank was supplied with the same system water that fish had previously been housed in, with sufficient aeration and heating. Fish were exposed to one of four treatments: (i) 0 μg/L VFX at 27°C; (ii) 1.0 μg/L VFX at 27°C; (iii) 0 μg/L VFX at 32°C; (iv) 1.0 μg/L VFX at 32°C; henceforth referred to as *Control*, *VFX*, *Temp*, and *VFX & Temp*, respectively. Fish were slowly acclimated to the temperature conditions over a 1-week period (∼0.7°C increase in temperature per day) prior to the start of the exposure. During the 1-week acclimation period, the tanks were equipped with back-hanging filtration units. The filters were removed when the acclimation period was over and the exposure period had begun. During the exposure period, the respective treatments mentioned above were dosed with 1.0 μg/L VFX (Millipore-Sigma-Aldrich, Oakville, ON, Canada). VFX aliquots dissolved in water were made in advance and stored at −20°C prior to daily dosing. The dosing protocol was adapted from an earlier study where similar static exposures were performed to examine the effects of VFX on neuroendocrine responses to stress in rainbow trout (*Oncorhynchus mykiss*; Melnyk-Lamont et al., 2014). The aforementioned study demonstrated that VFX concentrations are stable and are able to be maintained within the desired ranges throughout the duration of the exposure period.

Fish were fed ground flakes once daily until satiety, and 50% daily water changes were performed 1 h after feeding to remove waste and buildup of nitrogenous products. VFX daily dosing was performed at the same time water changes took place. Once a week, 100 mL water samples were collected from each tank at least an hour after the daily VFX dosing, and frozen at −20°C for later extraction and analysis of VFX concentrations using mass spectrometry. Fish mortality and tank temperature were monitored daily in addition to weekly water quality parameter-testing of ammonia, nitrate, nitrite, and pH. Throughout the duration of the experiment, there were no significant differences in mortality rates nor water quality parameters between treatments (Supplementary Tables 1, 2). Following the 21-day exposure period, feeding was ceased for 24 h and RMR was measured in a subset of fish. Another subset of fish was euthanized with an overdose of MS-222 (Millipore-Sigma-Aldrich, Oakville, ON, Canada; 0.5 g/L). Following euthanasia, fish lengths and weights were recorded and epaxial muscle was removed and snap frozen in liquid nitrogen and stored at −80°C for later analysis. The remainder of fish were used in concurrently occurring studies. Although fish were always acclimated for 1 week and exposed for 21 days, tanks had randomly staggered exposure start dates to facilitate the processing of physiological assays after the end of the exposure period.

### Routine Metabolic Rate

Routine metabolic rate was measured using a 170-mL glass swim tunnel respirometer equipped with a polymer optical fiber oxygen dipping probe, DAQ oxygen data acquisition system, Witrox oxygen reader, and AutoResp respirometry software (Loligo System, Tjele, Denmark). A water bath circulator equipped with a submersible water heater and a return pump controlled the temperature in the swim tunnel which was maintained at 27 or 32°C to match the exposure conditions. Throughout the experiment, the swim tunnel was programed to automatically cycle through three phases over 5 min: 60 s of flushing, 20 s of waiting, and 220 s of measuring oxygen concentration. Individual zebrafish were introduced into the swim tunnel and allowed to acclimate for up to an hour at a slow velocity of 5 cm/s to mimic normal activity in the exposure tanks and reduce spontaneous activity that would otherwise be observed in a static water chamber. A one-hour acclimation period was used to limit the depuration of VFX. Further, fish activity and oxygen consumption were continuously monitored during the acclimation and measurement periods, no significant changes in oxygen consumption nor fish activity were seen after the acclimation period. Following the acclimation period, up to six RMR measurements were taken over a period of 30 min. The mean of the three lowest metabolic rate measurements was used to calculate the metabolic rate for each individual. The average mass and total length of fish in each experimental group were as follows: *Control* (0.36 ± 0.02 g; 3.83 ± 0.04 cm); *VFX* (0.34 ± 0.03 g; 3.84 ± 0.09 cm); *Temp* (0.34 ± 0.02 cm; 3.78 ± 0.07 cm); *VFX & Temp* (0.32 ± 0.02 g; 3.70 ± 0.04 cm).

### Enzyme and Glucose Analysis

Frozen muscle tissue was powdered in liquid nitrogen using a mortar and pestle and 20–50 mg of tissue was homogenized in 20 volumes (20 × tissue mass) of extraction buffer (20 mM Hepes, 1 mM EDTA, and 0.1% Triton X-100, pH 7.0) using an electric homogenizer (Omni tissue homogenizer, Kennesaw, GA, United States). Sample homogenates were then centrifuged (12,000 *g*, 10 min, 4°C), and supernatants were used for enzyme assays. Enzyme activities were assayed in 96-well microplates using a Molecular Devices SpectraMax 190 spectrophotometer at assay temperatures matching those of exposure conditions at 340 nm unless stated otherwise. PK (E.C. 2.7.1.40), *LDH* (E.C. 1.1.1.27), and *HOAD* (E.C. 1.1.1.35) were assayed on fresh homogenates. Homogenates were then frozen as such at −80°C prior to the assays of *CS* (E.C. 2.3.3.1), *COX* (E.C. 1.9.3.1), and *CAT* (E.C. 1.11.1.6). Enzyme assays were performed following the protocols described in Mehdi et al. (2018). For reference, enzyme activities were also measured at assay temperatures matching both exposure temperatures (27–32°C), data shown in Supplementary Materials (Supplementary Figure 1). For glucose assay, muscle tissue samples were homogenized in two volumes of 8% perchloric acid and neutralized with 3 M K_2_CO_3_. Tissue glucose was measured following the standardized spectrophotometric protocol of Bergmeyer (1983) at 340 nm.

### Water Chemistry

A total 100-mL water samples were collected every 7 days from each tank and later analyzed to ensure VFX concentrations were maintained within nominal levels throughout the exposure experiment. Water samples were collected a minimum of 1 h after dosing and stored immediately at −20°C until extraction. One *VFX & Temp* (Day 7) sample broke during storage and was therefore excluded from analysis. VFX samples were quantified following (Rahman et al., 2010). Briefly, 100-mL samples were spiked with 100 μL [100 μg/L] deuterated VFX. Samples were then extracted using solid-phase extraction (SPE) in Oasis HLB cartridges (6 cc, 500 mg, Waters Corporation, Milliford, MA, United States). The eluents were collected in glass tubes and evaporated under a gentle stream of nitrogen gas, and then reconstituted in 500 μL methanol and stored at −20°C until analysis. Samples were then quantified using a Sciex API 3200 QTRAP LC-MS/MS system. The method detection limit (MDL) in a 500-mL sample was 1 ng/L. Since 100-mL samples were extracted in this experiment, the detection limit was calculated to be 5 ng/L based on the original MDL.

### Statistical Analysis

Data were analyzed using SigmaPlot 13.0 software (Systat Software Inc., San Jose, CA, United States). A two-way analysis of variance (ANOVA) was used to determine the main effects of VFX exposure (0–1.0 μg/L) and temperature exposure (27–32°C), as well as any interaction effects between the two stressors (VFX × temperature) on RMR, muscle enzyme activities, and glucose levels. Tukey’s *post hoc* test was used to identify any significant pairwise comparisons. A one-way ANOVA was used to identify significant differences between tank replicates in VFX concentrations. Data were log or square root transformed when necessary to meet the assumptions of normality. All data are presented as untransformed means ± standard error of the mean (SEM) and significant level was set to *P*-value < 0.05 for all tests.

## Results

### Water Chemistry and Temperature

**FIGURE 1:**
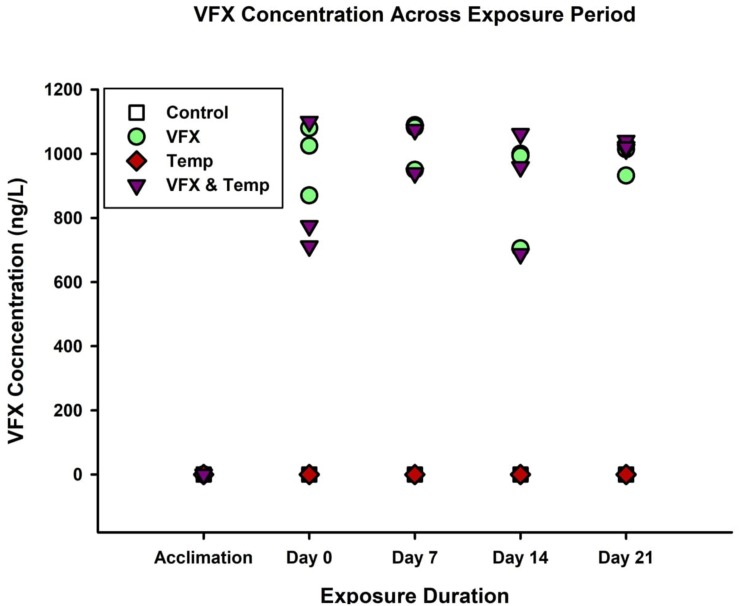
Venlafaxine concertation (ng/L) of each tank replicate within each treatment across the acclimation and exposure periods (Detection Limit = 5 ng/L).

Venlafaxine concentrations were below detection limit for the *Control* treatment (*n* = 12), 980.6 (±27.0) ng/L for the *VFX* treatment (*n* = 12), below detection limit for the *Temp* treatment (*n* = 12), and 965.2 (±59.3) ng/L for the *VFX & Temp* treatment (*n* = 11) during the exposure period (Figure 1). During the acclimation period, no VFX was detected in any of the tanks (*n* = 3 per treatment). Using a one-way ANOVA, no significant differences were detected between any of the tank replicates within each treatment, *P* = 0.509 for the *VFX* treatment and *P* = 0.281 for the *VFX & Temp* treatment. Similarly, water temperature in the exposure tanks was maintained at the intended temperatures throughout the entire duration of the experiment (7-day acclimation period and 21-day exposure period); *Control* treatment (27.6 ± 0.07°C), *VFX* treatment (27.1 ± 0.05°C), *Temp* treatment (32.3 ± 0.05°C), and *VFX & Temp* treatment (32.2 ± 0.06°C); actual temperature data is shown in Supplementary Table 1. As there were no significant differences in VFX concentrations and water temperatures between replicates within treatments, data were pooled from all three tank replicates for each treatment for statistical analysis.

### Routine Metabolic Rate

**FIGURE 2:**
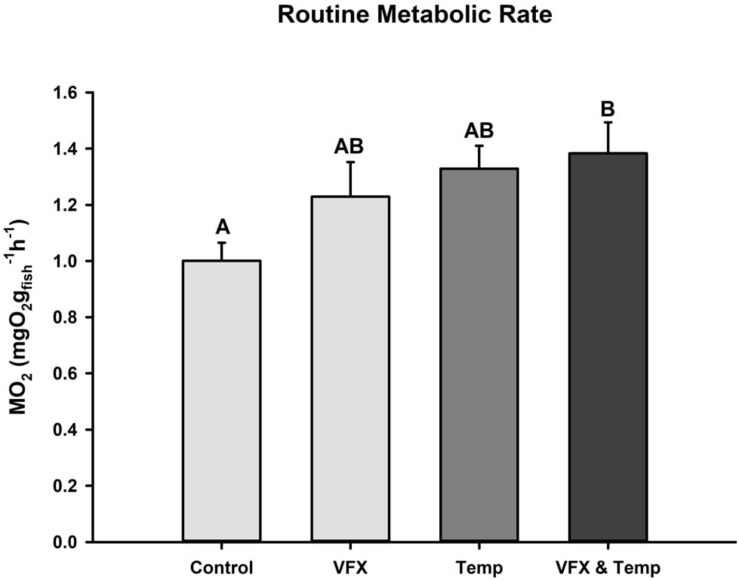
Routine metabolic rate measured in zebrafish 24 h post-exposure. RMR was highest in the multi-stressed group (*n* = 12) relative to *Control* (*n* = 12), but not VFX (*n* = 11) or Temp (*n* = 12). Effect of VFX: *F*_(__1_,_43__)_ = 2.063, *P* = 0.158; effect of temperature *F*_(__1_,_43__)_ = 6.014, *P* = 0.018; effect of VFX × temperature *F*_(__1_,_43__)_ = 0.782, *P* = 0.382 as per two-way ANOVA followed by Tukey’s *post hoc* test. Bars that do not share the same letters indicate significant differences.

Routine metabolic rate was significantly higher (∼38%) in the *VFX & Temp* group compared to the *Control*. RMR also tended to be higher in the *VFX* (∼23%) and *Temp* (∼33%) groups relative to *Control*, but these differences were not statistically significant (Figure 2).

### Muscle Glucose

**FIGURE 3:**
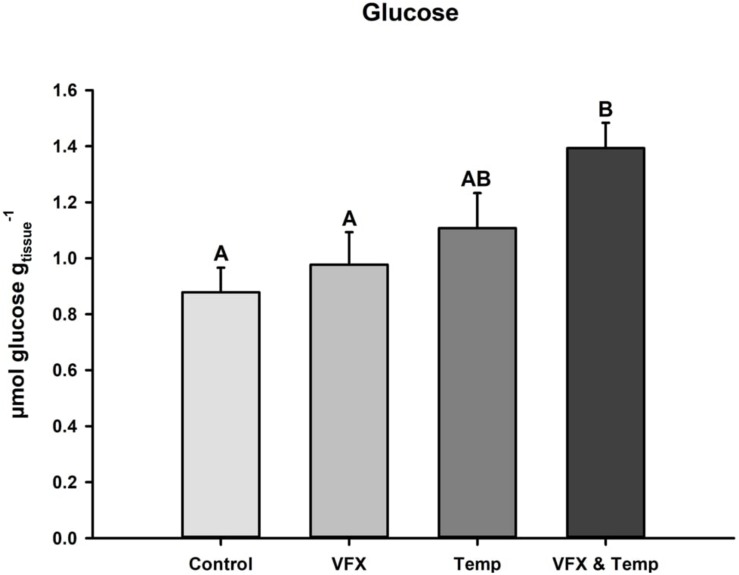
Glucose levels in muscle tissue of zebrafish post-exposure. Glucose levels were highest in *VFX & Temp* (*n* = 12) group compared to *Control* (*n* = 12) and *VFX* (*n* = 12), but not Temp (*n* = 12). Effect of VFX: *F*_(__1_,_44__)_ = 3.278, *P* = 0.077; effect of temperature *F*_(__1_,_44__)_ = 9.206, *P* = 0.004; effect of VFX × temperature *F*_(__1_,_44__)_ = 0.771, *P* = 0.385 as per two-way ANOVA followed by Tukey’s *post hoc* test. Bars that do not share the same letters indicate significant differences.

Muscle glucose concentrations after the 21-day exposure period are presented in Figure 3. Glucose levels were significantly higher in the *VFX & Temp* group compared to the *Control* and *VFX* groups, but not the *Temp* group.

### Enzyme Activities

**FIGURE 4:**
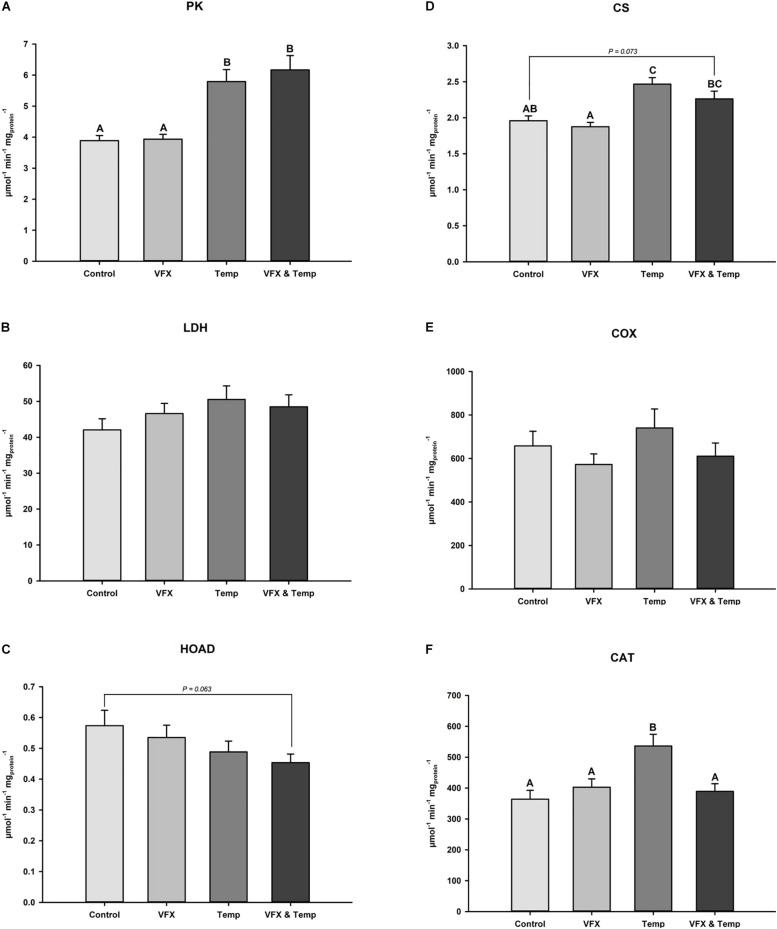
Muscle enzyme activity of **(A)** pyruvate kinase (PK), **(B)** lactate dehydrogenase (LDH), **(C)** 3-hydroxyacyl CoA dehydrogenase (HOAD), **(D)** citrate synthase (CS), **(E)** cytochrome c oxidase (COX), and **(F)** catalase (CAT) measured in male zebrafish post-exposure (*n* = 16–24 per treatment group). Bars that do not share the same letters indicate significant differences as per two-way ANOVA followed by Tukey’s *post hoc* test.

Activities of six muscle metabolic enzymes were measured at the respective exposure temperatures per treatment (Figure 4 and Table 1). PK activity was significantly higher in the *VFX & Temp* and *Temp* groups compared to the *Control* and *VFX* groups (Figure 4A). LDH activity was not significantly different across all treatments (Figure 4B). A significant temperature effect was observed in HOAD activity resulting in the *Temp* and *VFX & Temp* groups tending to be lower than the *Control* and *VFX* groups, but these pairwise comparisons were not statistically significant (Figure 4C). CS activity was significantly higher in the *Temp* group compared to the *Control*. CS activity was also significantly higher in the *VFX & Temp* group compared to the *VFX* group and tended (*P* = 0.073) to be higher than the *Control* group (Figure 4D). COX activity was not significantly different between treatments (Figure 4E). Finally, a significant interaction between VFX and temperature was demonstrated in CAT activity, where temperature induced an increase in CAT activity only when VFX was not present (Figure 4F).

**TABLE 1:** Fold changes in muscle enzyme activity among treatment groups relative to *Control* measured.

	**Fold change relative to *Control* group**				**Two-way ANOVA statistics**		
	***Control***	***VFX***	***Temp***	***VFX & Temp***	**Effect of VFX × temperature**	**Effect of VFX**	**Effect of temperature**
PK	−	1.01	1.49	1.59	*F*_(__1_,_84__)_ = 0.288; *P* = 0.593	*F*_(__1_,_84__)_ = 0.470; *P* = 0.495	***F*_(__1_,_84__)_ = 44.908; *P* < 0.001**
LDH	−	1.11	1.20	1.15	*F*_(__1_,_84__)_ = 0.971; *P* = 0.327	*F*_(__1_,_84__)_ = 0.144; *P* = 0.706	*F*_(__1_,_84__)_ = 2.420; *P* = 0.124
HOAD	−	0.93	0.85	0.79	*F*_(__1_,_82__)_ = 0.002; *P* = 0.966	*F*_(__1_,_82__)_ = 0.796; *P* = 0.375	***F*_(__1_,_82__)_ = 4.133; *P* = 0.045**
CS	−	0.96	1.26	1.15	*F*_(__1_,_84__)_ = 0.550; *P* = 0.460	*F*_(__1_,_84__)_ = 3.119; *P* = 0.081	***F*_(__1_,_84__)_ = 29.994; *P* < 0.001**
COX	−	0.87	1.12	0.93	*F*_(__1_,_80__)_ = 0.105; *P* = 0.747	*F*_(__1_,_80__)_ = 2.517; *P* = 0.117	*F*_(__1_,_80__)_ = 0.790; *P* = 0.377
CAT	−	1.11	1.47	1.07	***F*_(__1_,_84__)_ = 9.354; *P* = 0.003**	*F*_(__1_,_84__)_ = 3.163; *P* = 0.079	***F*_(__1_,_84__)_ = 6.798; *P* = 0.011**

*Significant main effects and interaction effects as per two-way ANOVA are bolded.*

## Discussion

In this study, we demonstrate that exposure to an environmentally relevant concentration of VFX in addition to a 5°C increase in water temperature can have several significant impacts on the metabolic responses of zebrafish. Most notably, VFX and temperature exposure had significant effects on RMR, various metabolic enzymes, and muscle glucose levels. These effects were most apparent when VFX and temperature were combined, however, several exceptions were observed. Studies often examine the effects of environmental perturbations using a single-stressor approach. However, this is the first study of its kind to look at the effects of both of these stressors (VFX and elevated temperature) individually and cumulatively using metabolic markers as sublethal endpoints under controlled lab conditions. At the whole-organism level, RMR tended to increase in all of the treatments relative to *Control*, but that was only significant between *VFX & Temp* and *Control*. This aligned well with our predictions, as metabolic rate is often positively correlated with temperature (Clarke and Fraser, 2004). Our results also indicate that VFX and temperature may be working cumulatively, as RMR was highest in the multi-stressed group. This is in line with other studies that have demonstrated the effects of various contaminants on metabolic rate, such as polychlorinated biphenyls, metals (McGeer et al., 2000), metals (Rajotte and Couture, 2002), and wastewater effluent (Du et al., 2018, 2019; Mehdi et al., 2018). At the tissue level, we demonstrated elevation in muscle glucose levels in the multi-stressed group compared to the *Control*, and increased PK activity in both *Temp* and *VFX & Temp* relative to *Control* and *VFX*. HOAD activity on the other hand tended to be lower in *Temp* and *VFX & Temp* relative to *Control*. CS activity was elevated in *Temp* relative to *Control* and VFX, but a similar effect was not observed in the *VFX & Temp* group. Finally, CAT activity was significantly higher in *Temp* relative to all other treatments. Although our study did not reveal overwhelming interaction effects between our two stressors of interest (VFX and temperature), we did demonstrate that VFX and temperature can have cumulative effects on several physiological parameters. The lack of strong interaction effects has been reported in a similar study examining the effects of a psychoactive pharmaceutical, oxazepam, and temperature on the behavior of European perch (*Perca fluviatilis*; Saaristo et al., 2019). It is also important to distinguish that most of the effects observed in our study were driven by temperature rather than VFX exposure. This is not surprising, as temperature is often regarded as an “ecological master factor” that impacts many of the endpoints that our study was interested in measuring. VFX, at environmentally relevant concentrations may not be potent enough to cause strong adverse effects in metabolic responses of fishes. This should be investigated further with a dose-dependent study looking at various VFX concentrations, possibly across multiple acclimation temperatures, rather than just two.

Exposure to anthropogenic contaminants poses a metabolic cost for fishes which can be further exasperated by other biotic and abiotic stressors, such as temperature (Craig et al., 2018). In this study we observed that carbohydrate metabolism was affected by increasing water temperature and VFX exposure, as indicated by the elevated muscle glucose levels and PK activity. This was expected, as energy demand is often higher at elevated environmental temperatures, and carbohydrates are the first energy reserves to be used (Hori et al., 2006; Pörtner and Knust, 2007). However, what was more intriguing was the fact that muscle glucose levels were only higher in the group exposed to both stressors, i.e., VFX and elevated temperature, suggesting that we are in fact observing a cumulative effect from both stressors, potentially causing fish to be energy deficient. A previous study has demonstrated similar results, where female rainbow darter (*Etheostoma caeruleum*) collected downstream of municipal WWTP in the Grand River watershed, ON, Canada had higher PK activity than their female counterparts collected from an upstream site (Mehdi et al., 2018). When observing the effects of VFX and temperature on the anaerobic capacity of zebrafish, we initially predicted that LDH activity, an enzyme involved in anaerobic glycolysis would be elevated in the exposed groups, however, our results were not supportive of this hypothesis. Our results are in line with previous studies demonstrating the lack of observable effects of VFX exposure and municipal WWTP effluent exposure on LDH activity in rainbow trout liver (Ings et al., 2011; Best et al., 2014). Our results indicate that neither environmentally relevant concentrations of VFX nor a 5°C increase in water temperature have significant effects on LDH activity, thereby, not allowing us to draw concrete conclusions about the effects of our two stressors on anaerobic glycolysis.

When measuring HOAD, an enzyme involved in β-oxidation of fatty acids and is commonly used as an indicator of lipid metabolism, a modest decrease was observed in activity in the groups that were exposed to the higher temperature, especially the *VFX & Temp* group. The increase in glucose levels and the opposing trends between PK and HOAD activities indicates that fish are more reliant on carbohydrate forms of energy production rather than lipid forms at the higher exposure temperature. The decrease in HOAD activity are comparable to a previous study that examined zebrafish reared at low and high temperatures, where it was found that HOAD activity was higher in zebrafish reared at lower temperatures (Schnurr et al., 2014). The acclimation response that we observed in groups exposed to higher temperatures could be maladaptive in fishes that experience frequent temperature fluctuations. It is known that metabolic demand increases in response to increasing water temperatures in ectotherms. Therefore, fish that are not able to plastically respond to these changes may be at a disadvantage, as they are unable to increase their lipolytic activity to make up for the increased metabolic costs of higher environmental temperatures, as observed by the increase in PK activity and glucose levels in the muscle. We also measured the activity of CS, a key enzyme in the citric acid cycle, which is often used as an indicator of aerobic capacity and potentially, mitochondrial abundance in the muscle (Rajotte and Couture, 2002; Lemos et al., 2003). We observed a general increasing trend in CS activity with the onset of both stressors (VFX and temperature); however, we only saw a significant increase in the *Temp* group compared to *Control* and *VFX*. We initially predicted that the onset of both stressors would result in more aerobic phenotypes, such as the increase in aerobic capacity and mitochondrial abundance as represented by elevation in CS activity. However, since this was only observed in the *Temp* group, it may suggest that VFX is indeed a metabolic disrupter and fish aren’t capable of adaptively responding to higher acclimation temperatures by increasing their aerobic capacity. This should be investigated further by subjecting multi-stressed fish to aerobically demanding challenges, such as critical thermal tolerance and critical swimming velocity tests. Finally, we assessed the effects of VFX and temperature on oxidative stress and capacity in the muscle tissue. We measured the activity of CAT, an enzyme involved in the defense against oxidative stress from reactive oxygen species (ROS) production, and is often used as an indicator of cellular damage and environmental stress (Atli et al., 2006; Kessabi et al., 2013). The increase in CAT activity in the *Temp* group was expected, as ectothermic metabolism often increases at higher temperatures, yielding more ROS and antioxidant enzyme activities would have to increase as well to combat these harmful byproducts of mitochondrial respiration (Davidson and Schiestl, 2001; Rocha et al., 2003; Lushchak and Bagnyukova, 2006; Bagnyukova et al., 2007). The increase in CAT activity in response to elevated water temperature is considered an adaptive response, indicating higher resistance to oxidative stress (Rudneva, 1999). However, this response was not present in fish exposed to both VFX and elevated water temperature, suggesting VFX could have a deleterious effect on the antioxidant defense mechanism and mitochondrial respiration, as has been demonstrated in isolated mammalian hepatocyte studies (Deavall et al., 2012; Ahmadian et al., 2016), although we did not see any significant impacts on COX activity, implying a more direct effect on CAT activity itself, which warrants further investigation. This observed interaction effect between temperature and VFX exposure on the antioxidant defense mechanism should be studied further, especially since fishes in the wild are exposed to a myriad of energetically demanding stressors and anthropogenic contaminants such as VFX, potentially causing them to be vulnerable to oxidative stress.

## Conclusion

In conclusion, this study aimed to explore effects of both VFX and elevated water individually and cumulatively on the metabolic physiology of zebrafish. This study also aimed to further explore how the toxicity of contaminants can change in the presence of other stressors, such as elevated temperatures. Many of the effects that were observed in our study are considered moderate, in the future, we will further investigate the costs that fish have to bare under multi-stressed conditions using fish with a narrower thermal tolerance than zebrafish, as well as using fish under different ontogenic stages. Physiological and ecological responses to multiple stressors can prove to be challenging and complex. However, multi-stressor research continues to be essential, as it aims to bridge the gap between laboratory and natural field settings, thereby, providing more accurate assessments than single-stressor research (Noyes et al., 2009; Ng et al., 2013).

## Data Availability Statement

The raw data supporting the conclusion of this manuscript will be made available by the authors, without undue reservation, to any qualified researcher.

## Ethics Statement

All experimental protocols followed the guidelines of the Canadian Council on Animal Care and were approved by the animal care committee at the University of Waterloo (AUPP #15-03).

## Author Contributions

HM was responsible for the designing and conducting the experiments as well as writing the manuscript. LB provided help in water chemistry analysis as well as providing many of the necessary equipment for this study. PC and MS provided extensive experimental design and editorial input to make this manuscript possible.

## Conflict of Interest

The authors declare that the research was conducted in the absence of any commercial or financial relationships that could be construed as a potential conflict of interest.
