# Supplementary material for: Multiple Stressors in the Environment: The Effects of Exposure to an Antidepressant (Venlafaxine) and Increased Temperature on Zebrafish Metabolism
Source: Front Physiol. 2019 Nov 19;10:1431. doi: 10.3389/fphys.2019.01431 (PMC6877669; doi:10.3389/fphys.2019.01431)
Supplement: Supplementary file 1 [file Data_Sheet_1.PDF]

Supplementary Material – Multiple Stressors in the Environment: The Effects of Exposure to an Antidepressant (Venlafaxine) and Increased Temperature on Zebrafish Metabolism

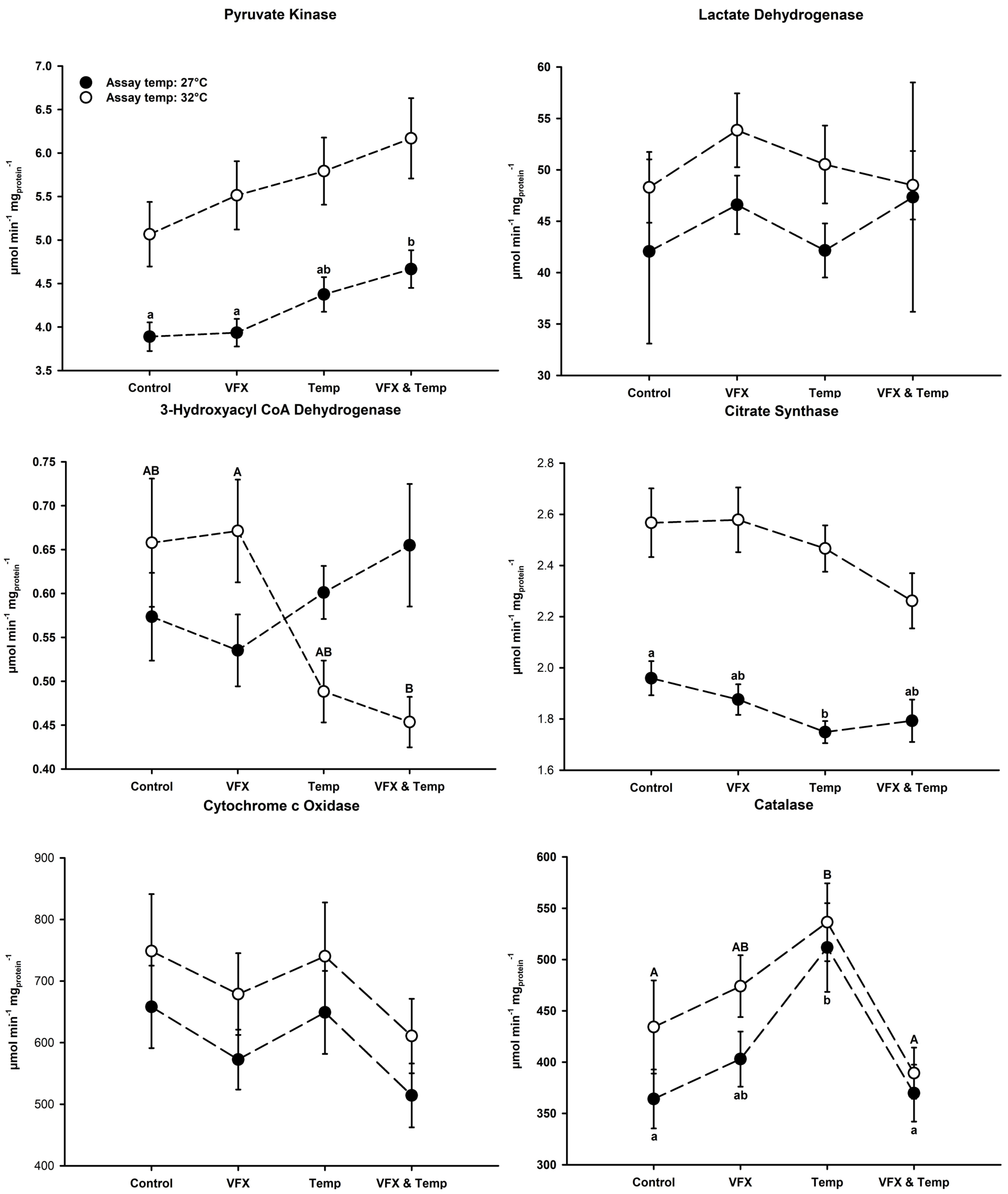

Supplementary Figure 1: Muscle enzyme activity of pyruvate kinase (PK), lactate dehydrogenase (LDH), 3-hydroxyacyl CoA dehydrogenase (HOAD), citrate synthase (CS), cytochrome c oxidase (COX), and catalase (CAT) measured in male zebrafish post-exposure (n = 16-24 per treatment group). Enzyme activity was measured at two assay temperatures 27°C and 32°C, presented as black and white circles, respectively. Circles that do not share letters indicate significant differences in enzyme activity, lowercase letters were reserved to show differences in enzyme activities when measured at 27°C and uppercase letters when measured at 32°C.

frontiers

Supplementary Table 1. Daily temperature values for each tank across the exposure period measured by a digital thermometer.

Timeline	Control 1	Control 2	Control 3	VFX 1	VFX 2	VFX 3	Temp 1	Temp 2	Temp 3	VFX & Temp 1	VFX & Temp 2	VFX & Temp 3
Exposure Day 0	28.1	28.3	26.9	27.3	28.3	27.5	32.5	31.8	32	32.3	31.4	32.2
Exposure Day 1	27.7	27.9	28.1	27.1	28.7	27.7	32.3	32.6	31.8	32.1	32.5	32
Exposure Day 2	27.4	27.9	27.6	27.5	27.6	27	32.1	32.8	32.4	32.9	31.9	32.4
Exposure Day 3	28.1	28.1	27.3	27.4	27.3	27.1	31.9	32.8	31.3	31.8	31.5	32.1
Exposure Day 4	27.2	27.9	27.5	26.9	27.3	26.5	31.9	32.6	31.5	31.9	32.2	31.9
Exposure Day 5	28.1	27.6	27.2	27.4	27.4	26.8	32.9	32.5	32.8	32.6	32.8	31.3
Exposure Day 6	27.8	27.9	26.7	27.3	27.4	27.3	32.1	32.7	31.5	32.7	32.5	31.7
Exposure Day 7	28.2	28.5	27.1	27.5	26.8	26.6	32.1	32.5	32.2	31.8	32.4	32.2
Exposure Day 8	28.3	28.1	27.1	27.1	26.9	27	32.3	32.7	31.5	32.6	33	32
Exposure Day 9	27.7	27.9	26.1	27.1	27	26.9	32.2	32.2	31.9	32.4	32.3	32.7
Exposure Day 10	27.4	28.3	27.2	27.1	27	26.7	32.4	32.6	32.1	32.4	32.9	32.5
Exposure Day 11	27.3	28.3	27.1	27.1	26.9	26.8	31.9	32.8	32.4	31.8	32	32.9
Exposure Day 12	27.3	28.1	26.8	27	26.9	27.2	32	32.7	32.7	31.9	31.5	31.7
Exposure Day 13	27.7	28.1	26.6	27.1	26.7	27.6	32.2	32.6	32.6	32.8	31.8	32
Exposure Day 14	27.9	28.5	27.1	27	26.8	27.6	32.5	32.6	32.4	31.6	32.1	32.2
Exposure Day 15	27.5	28.1	26.6	26.9	26.9	26.9	31.7	32.7	32.2	32.2	31.6	32.8
Exposure Day 16	27.5	28.1	27.1	26.6	26.9	26.9	32	32.6	32.2	32.7	32.9	32.4
Exposure Day 17	28.1	28.5	27.1	26.5	26.7	27	32.3	32.7	32.8	31.8	32.8	32.4
Exposure Day 18	28.2	27.9	27.1	26.7	26.8	27	32	32.2	31.9	31.8	32.1	32.1
Exposure Day 19	27.6	28.1	26.8	27	26.8	27.2	31.8	32.7	32.1	32.3	33.1	31.9
Exposure Day 20	27.9	27.9	26.6	27	26.9	27	32.6	32.8	32.1	31.9	32.8	32.1
Exposure Day 21	28.1	27.9	26.9	27.2	26.7	26.8	32.3	32.6	31.9	32.4	32.7	32.2
Mean	27.8	28.1	27.0	27.1	27.1	27.1	32.2	32.6	32.1	32.2	32.3	32.2
S.E.M	0.1	0.1	0.1	0.1	0.1	0.1	0.1	0.1	0.1	0.1	0.1	0.1

Supplementary Table 2. Daily mortality observations for each tank across the exposure period.

Timeline	Control 1	Control 2	Control 3	VFX 1	VFX 2	VFX 3	Temp 1	Temp 2	Temp 3	VFX & Temp 1	VFX & Temp 2	VFX & Temp 3
Exposure Day 0	0	0	0	0	0	0	0	0	0	0	0	0
Exposure Day 1	0	0	0	0	0	0	0	0	0	0	0	0
Exposure Day 2	0	0	0	0	0	0	0	0	0	0	0	0
Exposure Day 3	0	0	0	0	0	0	0	0	0	0	0	0
Exposure Day 4	0	0	0	0	0	0	0	0	0	0	0	0
Exposure Day 5	0	0	0	0	0	0	0	0	0	0	0	0
Exposure Day 6	0	0	0	0	0	0	0	0	0	0	0	0
Exposure Day 7	0	0	1	0	0	0	0	0	0	0	0	0
Exposure Day 8	0	0	0	0	0	0	0	0	0	0	0	0
Exposure Day 9	0	0	0	0	0	0	0	0	0	0	0	0
Exposure Day 10	0	0	0	0	0	0	1	0	0	0	0	0
Exposure Day 11	0	0	0	0	0	0	0	0	0	0	0	0
Exposure Day 12	0	0	0	0	0	0	0	0	0	0	0	0
Exposure Day 13	0	0	0	0	0	0	0	0	0	0	0	0
Exposure Day 14	0	0	0	0	0	0	0	0	0	0	0	0
Exposure Day 15	0	0	0	0	0	0	0	0	0	0	0	0
Exposure Day 16	0	0	0	0	0	0	0	0	0	0	0	0
Exposure Day 17	0	0	0	0	0	0	0	0	0	0	0	0
Exposure Day 18	0	0	0	0	0	0	0	0	0	0	0	1
Exposure Day 19	0	0	0	0	0	0	0	0	0	0	0	0
Exposure Day 20	0	0	0	0	0	0	0	0	0	0	0	0
Exposure Day 21	0	0	0	0	0	0	0	0	0	0	0	0
